# Dichroism-sensitive photoacoustic imaging for in-depth estimation of the optic axis in fibrous tissue

**DOI:** 10.1016/j.pacs.2024.100676

**Published:** 2024-12-09

**Authors:** Camilo Cano, Amir Gholampour, Marc van Sambeek, Richard Lopata, Min Wu

**Affiliations:** a Department of Biomedical Engineering, Eindhoven University of Technology, De Rondom 70, Eindhoven, The Netherlands; b Department of Vascular Surgery, Catharina Ziekenhuis Eindhoven, Michelangelolaan 2, The Netherlands

**Keywords:** Photoacoustic imaging, Dichroism imaging, Anisotropic tissue, Optic axis estimation

## Abstract

Photoacoustic imaging (PAI) is a developing image modality that benefits from light–matter interaction and low acoustic attenuation to provide functional information on tissue composition at relatively large depths. Several studies have reported the potential of dichroism-sensitive photoacoustic (DS-PA) imaging to expand PAI capabilities by obtaining morphological information of tissue regarding anisotropy and predominant orientation. However, most of these studies have limited their analysis to superficial scanning of samples, where fluence effects are negligible. Herein, we present a mathematical model for the in-depth analysis of the DS-PA signal of biological samples, focusing on estimating tissue orientation. Our model is validated with a B-scan setup for DS-PA imaging in ex-vivo porcine tendon samples, for which collagen displays optical anisotropy. Results show that for in-depth DS-PA imaging, the accumulative fluence modulation due to dichroism overcomes the effect of absorption dichroism affecting the measured signals; however, this effect can be corrected based on the presented model for determining fiber orientation.

## 1 Introduction

Photoacoustic imaging (PAI) is a developing hybrid image modality with multiple valuable applications in medical diagnosis [1], [2], [3]. In PAI, the initial pressure signal is generated by local thermoelastic expansion resulting from the optical absorption of pulsed radiation, which is subsequently measured using standard ultrasound transducers [4]. Thanks to the high wavelength-dependent optical absorption contrast between different tissues, PAI offers valuable functional/compositional information while leveraging the low acoustic attenuation characteristic of ultrasound imaging [5], [6], [7], enabling tissue characterization at depths beyond the reach of conventional optical imaging techniques and with high spatial resolution [4], [8], [9].

In addition to wavelength-dependent optical absorption contrast, some structurally anisotropic tissues like collagen fibers show optical absorption anisotropy, meaning that the optical absorption changes with the polarization of light [10], [11], [12]. This direction-dependent variation of the absorption is known as dichroism, and it is frequently linked with the structural and functional properties of the fibrous tissue [11]. Its analysis can be beneficial for many different applications, for instance, monitoring collagen restoration of burn wounds [13], assessing the orientation of myocardial fibers in the heart and its valves [14], [15], tracking the remodeling of collagen fibers in vessels due to atherosclerosis [16], and characterizing structural integrity of tendons and cartilages [17], [18]. In 2018, Qu et al. [19] proposed a dichroism-sensitive photoacoustic (DS-PA) computed tomography method that proved the potential of the technique to detect the orientation of the optic axis in uniaxial dichroic tissues, even while embedded in turbid media. As a pure optical imaging technique, polarization-sensitive optical coherence tomography has provided promising results in detecting the collagen fiber structure of the cornea and sclera [20]. However, the limited penetration depth of this technique (∼1–2 mm [21], [22]) restricts its application in biomedical imaging. Therefore, recently many studies are ongoing to further explore the feasibility of dichroism-sensitive photoacoustic imaging (DS-PAI) to characterize tissue anisotropy [19], [23], [24], [25]; however, these studies predominantly focus on superficial scanning, where the effect of fluence is neglectable.

To broaden the applicability of the DS-PA technique, in-depth anisotropy measurement is paramount, allowing two-dimensional imaging of geometry and tissue structure. However, this requires further analysis of the effect of build-up fluence modulation due to the dichroism. Therefore, we developed a DS-PAI model for in-depth detection of fiber orientation in biological tissue. We expand the model by considering the DS-PA signals as the product of the local fluence modulation with the absorption modulation. We propose a method to estimate the optic axis orientation and fluence parameters by minimizing the error between the measured DS-PA signals and our model. We evaluated our model by imaging through porcine tendons to a depth of 3.2 mm.

## 2 Materials and methods

### 2.1 DS-PAI model

We consider an anisotropic fibrous tissue with a dominant, uniaxial fiber direction, where its optic axis is aligned with the fibers’ orientation. The sample is illuminated with linearly polarized light perpendicular to the fibers to obtain the maximum dichroism effect. Furthermore, the state-of-the-art indicates that given the magnitude of the dichroism and birefringence in tendons [26], it is appropriate to neglect significant variations in the orientation of the main component of light polarization as it travels through the sample (see Appendix A). In this study, we will assume a scattering-free medium to simplify the formulation of the model. Under the aforementioned conditions, the photoacoustic pressure p can be defined as:

(1) $$p\left(\vec{r},\theta \right)=\Gamma \eta_{th}F\left(\vec{r},\theta \right)\mu_{a}\left(\vec{r},\theta \right),$$

where Γ is the Grueneisen parameter (dimensionless) assumed to be spatially invariant, $\eta_{th}$ is the constant of energy conversion efficiency (dimensionless), F is the light fluence (in [J/cm${}^{2}$]), $\mu_{a}$ is the absorption coefficient (in cm^−1^) at location $\vec{r}$, and θ is the orientation of the polarization plane (in [rad]). For a beam propagating in an absorbing anisotropic tissue, the absorption coefficient is described as [27]:

(2) $$\mu_{a}\left(\vec{r},\theta \right)={\bar{\mu }}_{a}\left(\vec{r}\right)+\frac{\Delta \mu_{a}\left(\vec{r}\right)}{2}\cos\left(2\left(\theta -\phi \left(\vec{r}\right)\right)\right),$$

where ϕ is the orientation of the optic axis of the tissue. ${\bar{\mu }}_{a}\left(\vec{r}\right)=\frac{\mu_{a,o}\left(\vec{r}\right)+\mu_{a,e}\left(\vec{r}\right)}{2}$ is the mean absorption coefficient between the ordinary ($\mu_{a,o}$, perpendicular to the optic axis) and extraordinary ($\mu_{a,e}$, parallel to the optic axis) components of the absorption, and $\Delta \mu_{a}\left(\vec{r}\right)=\mu_{a,o}\left(\vec{r}\right)-\mu_{a,e}\left(\vec{r}\right)$ is the difference between components. To describe discrete measurements at different polarization angles, we redefine $\theta =\omega_{0}t$, where $\omega_{0}$ is the angular sampling of the acquisitions and t is a measurement counter. The fluence in such a dichroic tissue without scattering is defined by:

(3) $$F\left(\vec{r},\theta \right)=F_{0}e^{\left(-\int_{\ell }\mu_{a}\left(\vec{r},\theta \right)dr\right)},$$

where the initial fluence $F_{0}$ is equal for all different polarizations, and dr corresponds to the differential of length as the light travels through the path ℓ. After substituting Eq. (2) into Eq. (3), expanding it into Maclaurin series and truncating at the second term, Eq. (3) can be approximated to:

(4) $$F\left(\vec{r},t\right)\approx F_{0}e^{\left(-\int_{\ell }{\bar{\mu }}_{a}\left(\vec{r}\right)d\vec{r}\right)}\left[1+\int_{\ell }\frac{\Delta \mu_{a}\left(\vec{r}\right)}{2}\cos\left(2\left(\omega_{0}t-\phi \left(\vec{r}\right)\right)\right)d\vec{r}\right].$$

After plugging Eqs. (2), (4) into Eq. (1), the local photoacoustic pressure in an anisotropic media can be defined as the product of two sinusoidal functions:

(5) $$p=\bar{p}\left(\left[1+\alpha \cos\left(2\omega_{0}t-2\psi \right)\right]\left[1+\beta \cos\left(2\omega_{0}t-2\phi \right)\right]\right),$$

where $\bar{p}$ corresponds to the mean PA signal independent of polarization, α is the local fluence modulation, ψ is the local phase of the fluence, and β describes the ratio between the linear dichroism and mean absorption of the tissue.

(6) $$\bar{p}=\Gamma \eta_{th}{\bar{\mu }}_{a}\left(\vec{r}\right)F_{0}e^{\left(-\int_{\ell }{\bar{\mu }}_{a}\left(\vec{r}\right)d\vec{r}\right)}.$$

$$\beta =\frac{\Delta \mu_{a}\left(\vec{r}\right)}{2{\bar{\mu }}_{a}\left(\vec{r}\right)}.$$

$$\alpha \cos\left(2\omega_{0}t-2\psi \right)=\int_{\ell }\frac{\Delta \mu_{a}\left(\vec{r}\right)}{2}\cos\left(2\left(\omega_{0}t-\phi \left(\vec{r}\right)\right)\right)d\vec{r}.$$

Eq. (5) can be expanded and grouped as the sum of a continuous component(DC), the fundamental frequency (H1) corresponding to $\omega_{0}$, and a second harmonic (H2):

(7) $$p=p_{\mathrm{DC}}+p_{\mathrm{H1}}+p_{\mathrm{H2}},$$

where

(8) $$p_{\mathrm{DC}}=\bar{p}\left(1+\frac{\alpha \beta \cos\left(2\left(\phi -\psi \right)\right)}{2}\right),$$

$$p_{\mathrm{H1}}=\bar{p}\left(\beta \cos\left(2\phi -2\omega_{0}t\right)+\alpha \cos\left(2\psi -2\omega_{0}t\right)\right),$$

$$p_{\mathrm{H2}}=\bar{p}\left(\frac{\alpha \beta \cos\left(2\left(\phi +\psi -2\omega_{0}t\right)\right)}{2}\right).$$

Since we mainly rely on processing the fundamental frequency of the DS-PA signal, we will limit our analysis accordingly. The absolute value of the Fourier transform at the fundamental frequency is:

(9) $$\|ℱ\left\{p_{\mathrm{H1}}\right\}\|=\bar{p}\pi \sqrt{\alpha^{2}+\beta^{2}+2\alpha \beta \cos\left(2\phi -2\psi \right)}.$$

It depends on the contributions of the fluence and absorption modulations over the mean PA signal. After dividing Eq. (9) by the $\bar{p}$ to remove the characteristic exponential decrease in amplitude due to fluence, we can derive relevant information from the sample. For example, if this ratio remains constant as light travels, we can infer there is no dichroism regardless of any sinusoidal modulation in the DS-PA signal since β=0 and α stays constant at the accumulated fluence modulation. The phase of the fundamental frequency is:

(10) $$∠ℱ\left\{p_{\mathrm{H1}}\right\}=\tan^{-1}\left(\frac{\alpha sin\left(2\psi \right)+\beta sin\left(2\phi \right)}{\alpha cos\left(2\psi \right)+\beta cos\left(2\phi \right)}\right).$$

**Fig. 1:**
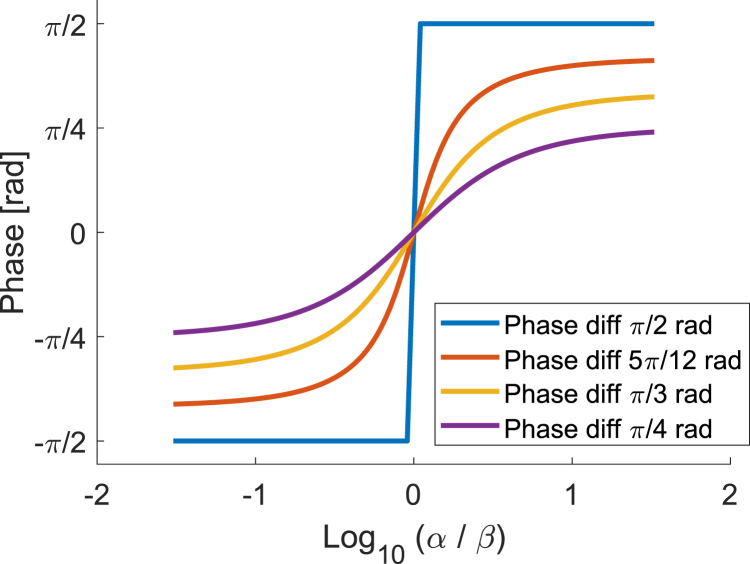
Plot of Eq. (10). The function behaves as a sigmoid function with asymptotes at the phases of the absorption (left) and the fluence (right). The phase of the DS-PA signal will depend on the difference between phases and the ratio between fluence and absorption modulations α/β.

Eq. (10) resembles the behavior of a sigmoid function, where the phase changes between ϕ and ψ depending on the ratio between α and β. Fig. 1 illustrates such a behavior.

This implies that for a constant phase difference, the DS-PA phase varies between the phase of the fluence and absorption and changes smoothly with α. Since the fluence phase and amplitude can only change smoothly, abrupt changes in the DS-PA phase are due to variations in the optics axis orientation (ϕ). For the particular case of a homogeneous sample, where ϕ=ψ+π/2, the model indicates that the photoacoustic phase will be initially aligned with the optic axis of the fibers and experience an abrupt phase shift of π rad when the fluence modulation overcomes the dichroism of the tissue (α>β). To consider the effect of scattering in the model, the optical absorption $\mu_{a}$ must be replaced by the effective attenuation of the signal in Eq. (3). This will change the magnitude of the parameters in all the equations, but the obtained relations remain valid for the propagation of straight beams.

### 2.2 Experiment description

**Fig. 2:**
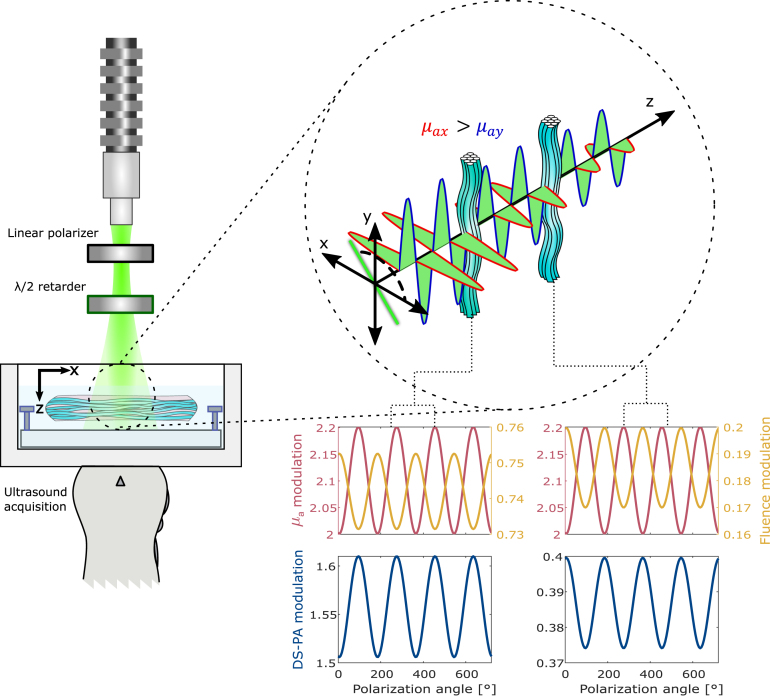
Schematic of the DS-PA imaging system and working principle. The light source is linearly polarized, and the plane of polarization is controlled using a λ/2 waveplate. The tissue absorbance will change for the orthogonal components of the electric field of light with respect to the sample orientation. By changing the plane of polarization of light, the PA signal acquired by the ultrasound probe will have a sinusoidal modulation. The DS-PA signal varies in amplitude and phase depending on the local fluence and absorption.

To analyze the in-depth modulation of DS-PA signals from fibrous tissue as a function of light polarization, we employed a transmission mode PA dichroism-sensitive imaging system as depicted in Fig. 2. We used a tunable pulsed optical parametric oscillator laser (OPOTEK radiant HE 355 LD, Carlsbad-California, USA) tuned at 532 nm with a pulse repetition rate of 10 Hz for illumination. The light was delivered to the sample using a custom-made fiber bundle (CeramOptec, Bonn, Germany) with an aperture of 5 mm, with a fluence of 5 mJ/cm${}^{2}$ on top of the sample. A linear polarizer placed in front of the fiber bundle polarizes the light. It was followed by a λ/2 waveplate retarder, which was rotated by a motorized rotation stage (K10CR1/M, Thorlabs, Newton, New Jersey, United States) to control the polarization angle. Acoustic pressure was measured using a Verasonics Vantage 256 system with a linear array probe at the center frequency of 7.8 MHz (L11-5, Kirkland-Washington, USA). The system generates B-mode images with an axial resolution of 0.3 mm and a lateral resolution of 0.5 mm. The sample was placed in a water tank (the bottom of the tank was sealed using a thin film), with perpendicular illumination from the top and US receiving from the bottom as depicted in Fig. 2.

We validated our method on porcine tendons, since they have a substantial dichroism at 532 nm [26]. Tendon samples are mounted under tension to ensure collagen fibers are aligned. We first performed the measurements on a single porcine tendon and then on two stacked tendons with an angle between them. Fig. 2 illustrates the expected behavior of the DS-PA signal in a homogeneous tendon sample. The detected DS-PA signal exhibits sinusoidal modulation, with its phase determined by the phases of the absorption coefficient ($\mu_{a}$) and the fluence, as well as their respective amplitudes, in accordance with Eq. (10). At the first interaction with the tissue, the normalized modulation of $\mu_{a}$ ($\Delta \mu_{a}/{\bar{\mu }}_{a}$ ∼0.1) is greater than that of the fluence (∼0.03), causing the DS-PA signal to align with the absorption phase. As light propagates further, the fluence modulation increases (∼1.6), eventually dominating the signal and shifting the DS-PA signal phase towards the fluence, despite no change in the fiber orientation ($\mu_{a}$ phase). This observation underscores the importance of accurately assessing DS-PA signals.

**Fig. B.8:**
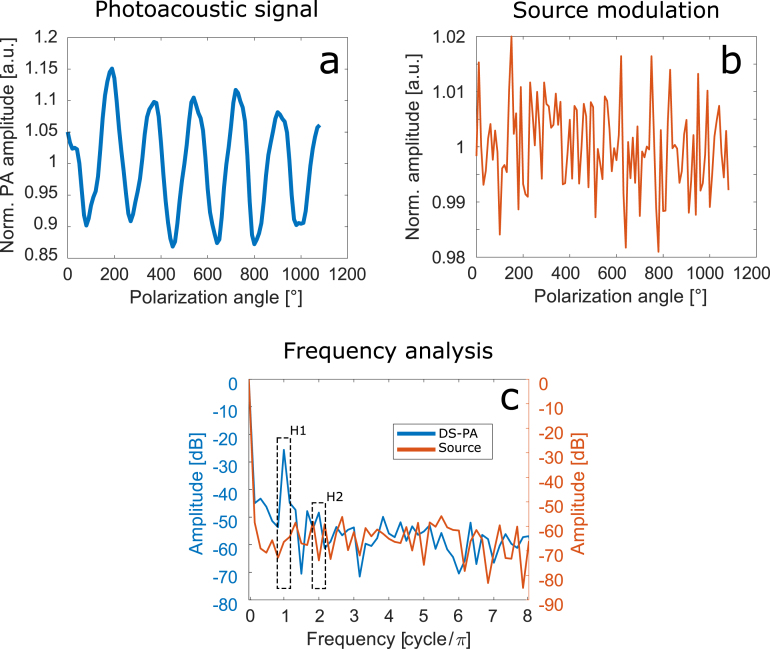
Example of DS-PA signal. (a) is a representative DS-PA signal at a depth of 1 mm. (b) is the normalized source modulation, with a mean energy of 5 mJ. (c) is the Fourier spectrum of the signals, where *H1* and *H2* are the location of the fundamental frequency and second harmonic of the signal.

We acquired 109 datasets per sample as we changed the polarization angle from 0° to 1080° (three revolutions) in steps of 10°. These scan settings were selected to obtain enough points to sample the sinusoidal modulation of the DS-PA signals (Fig. 2) and account for noise and laser instability. To enhance the signal-to-noise ratio (SNR), each photoacoustic image (PAI) was averaged 40 times. A built-in spectrometer recorded the laser pulse energy to correct fluence variation (see Fig. B.8). Additionally, co-registered ultrasound images were acquired using plane-wave imaging with 31 steering angles for a range of ±18°. All the data were reconstructed using delay-and-sum beamforming.

### 2.3 Data analysis

To estimate the orientation of the fibers, we calculate the fast Fourier transform (FFT) of the DS-PAI data acquired at the different polarization angles. In general, each pixel within the sample will have three relevant frequency components: the DC of the PA signal, the fundamental frequency corresponding to the number of cycles in the signal per rotation of the polarization plane by the λ/2 retarder, and the second harmonic originated from the product of the absorption and fluence sinusoidal modulations. We focus our analysis on the fundamental frequency component since its signal has a sufficient SNR throughout the sample.

We use the amplitude of the fundamental frequency component described by Eq. (9) to determine whether the signal modulation is large enough to obtain a reliable estimation. We use the Rose criterion [28] to mask noise signals when the modulation at a pixel is less than five standard deviations of the background noise. However, an exception applies when the signal decreases at the same position that the phase shows a variation of π/2, as it corresponds to a unique scenario when the absorption (β) and fluence (α) modulations have the same amplitude but are in counter-phase. After estimating the tissue optic axis, we employ linear interpolation on the masked areas.

The detected DS-PA phase is governed by the sigmoid function presented in Eq. (10); therefore, the signal phase will vary between the fluence (ψ) and absorption (ϕ) phases. Considering that our samples have homogeneous dichroism, we assume that β is constant or its variations are negligible. To determine the orientation of the optical axis ϕ, we estimate the parameters α, ψ, and ϕ by minimizing the objective function:

(11) $$ɛ={\left(\eta -\tan^{-1}\left(\frac{\alpha \sin\left(2\psi \right)+\beta \sin\left(2\phi \right)}{\alpha \cos\left(2\psi \right)+\beta \cos\left(2\phi \right)}\right)\right)}^{2}+$$

$${\left(\xi -\sqrt{\alpha^{2}+\beta^{2}+2\alpha \beta \cos\left(2\phi -2\psi \right)}\right)}^{2},$$

where η is the experimental phase of the first harmonic and ξ is the experimental amplitude of the first harmonic divided by $\pi \bar{p}$ (mean PA pressure). Since we have an underdetermined system, we solve the problem by constraining the seed and boundaries of the parameters for some particular solutions of Eqs. (9), (10). Additionally, as α and ψ evolve with the fluence build up, we estimate the parameters sequentially as the light travels through the sample. The proposed approach is presented in Algorithm 1:

**Figure:**
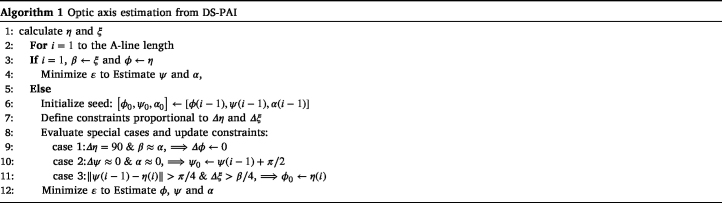

Depending on the contribution of the local fluence modulation, four particular scenarios are taken into consideration. In the first layer (surface), fluence modulation is negligible; therefore, ϕ=η and β=ξ. The second consideration is that a phase jump of π/2 when α≈β corresponds to a particular solution of Eq. (10) and ϕ should not change significantly. Additionally, if α=0 after the first surface of the sample, the fluence phase will experience a π/2 phase shift. The final consideration is that both α and ψ should change in a continuous fashion; therefore, sudden simultaneous variations of η and ξ are due to the change of ϕ. Based on this restriction, we optimize the seed and constrain the solution space for minimization. The parameters are estimated using the MatLAB® function fmincon, employing the sequential quadratic programming solver. We use the first order optimality measure as stopping criteria with the default convergence tolerance of $1\cdot 10^{-6}$ [29].

## 3 Results

### 3.1 Analysis on porcine tendon: single layer

**Fig. 3:**
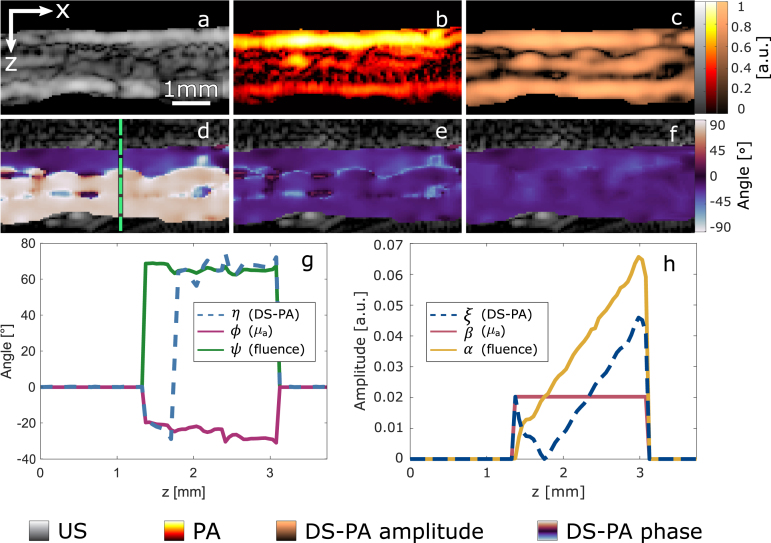
Imaging of porcine tendon with DS-PAI. (a) shows the normalized ultrasound imaging, (b) the mean PA imaging, and (c) the normalized amplitude of the DS-PAI modulation. (d) is the phase of the DS-PAI, (e) is the estimated fibers’ orientation ϕ, and (f) is the final estimation after interpolating areas with low modulation. (g) illustrates a single A-line profile (dashed line in d) for DS-PA, $\mu_{a}$, and the fluence phases. (h) shows the amplitude of the signal modulation for DS-PA, $\mu_{a}$, and the fluence.

We begin validating the model on a porcine tendon for the case of a homogeneous tissue. Fig. 3a and b show the ultrasound and mean photoacoustic images (averaged PA images acquired at all different polarization angles) of the cross-section of the tendon. Fig. 3c is the normalized modulation of the first harmonic. Dark pixels correspond to the locations where signal modulation is too low to accurately determine the phase of the PA signal due to bandwidth limitations or the cancellation of the fluence and absorption signals. Fig. 3d shows the phase of the DS-PA signal. The change of 90° in the detected phase is due to the fluence modulation overcoming the absorption modulation. Fig. 3e shows the estimated orientation of the optical axis from the DS-PAI phase and amplitude. The pixels with a low modulation are finally masked and interpolated in Fig. 3d. For the A-line indicated in Fig. 3d, g shows the estimated phases for the fluence (ψ) and absorption (ϕ) at different depths (z), and Fig. 3h presents the respective modulations α and β. It can be observed that at the point at which the fluence modulation α reaches the dichroism value β, the DS-PA phase will switch from ϕ to ψ. Similarly, the amplitude of the DS-PA signal will be null at this point since the fluence and absorption signals will cancel because, in a homogeneous media, they will be in counter-phase.

**Fig. 4:**
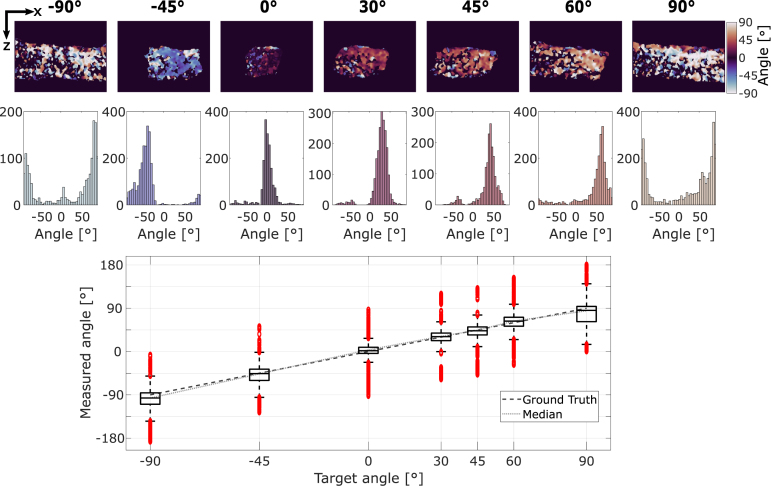
Porcine tendon imaged with DS-PAI at different orientations. The top row shows the measured fiber orientation using DS-PAI for various orientations of the tendon between −90° and 90°. The second row shows the corresponding histograms. The third row shows the box-and-whisker plot for each image, indicating a linear correlation between the tendon orientation and the measured angle.

To assess the accuracy of our method to track the fibers’ orientation, we imaged the tendon rotated at different angles and determined the orientation of the fibers with DS-PAI. The top row of Fig. 4 shows the cross-section images of the tendon obtained at different orientations masked by the modulation to remove low SNR signals. The second row shows the histograms describing the distribution of the estimated fiber’s orientation, where the peak of the distribution indicates the average orientation of the collagen fibers in the tendon. The bottom row shows the box-and-whisker plots of the measured angle for each orientation of the tendon. We can clearly see the linear correlation between the true orientation and the estimated angles in a homogeneous sample. The mean square error of the orientation detection is 3.97°.

### 3.2 Analysis on porcine tendon: stacked layers

**Fig. 5:**
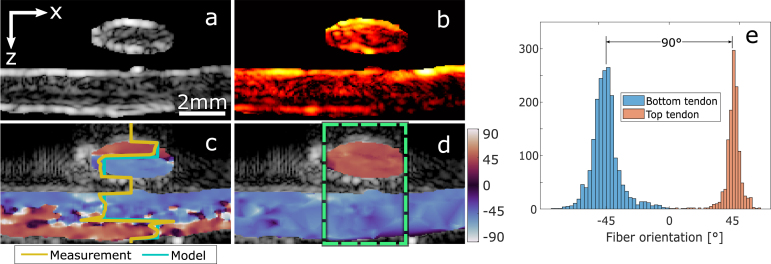
Analysis of stacked tendons perpendicularly oriented. (a) is the Ultrasound image, and (b) is the mean PA image. (c) is the phase detection for the two layers. The profile plot shows the behavior of the DS-PA phase along with a model-based profile of a perfectly homogeneous tissue for visualization. (d) is the corrected estimation of the fibers’ orientation. (e) shows the histograms for the two tendons within the region indicated by the dashed square. The peak of the distributions indicates the mean fiber orientation for the tendon.

To explore the possibility to characterize the fiber’s orientation in a non-homogeneous sample, we have performed two different measurements. The first one is done with two stacked tendons oriented perpendicular to each other. Fig. 5a,b show the ultrasound and PAI images of the tendons. There is a small gap between the tendons, which we overlook assuming that the effect of water in the polarization is negligible; however, the change in media introduces a significant drop in signal that does not affect the detected phase but reduces its SNR. Fig. 5c shows the detected phase map of the fundamental frequency. The profiles of the detected phase and the model-based phase are plotted at the center of the image, illustrating how the phase only changes in step variations since ϕ and ψ are always in phase or counter-phase. Like in a single tendon case, a step change in phase happens in both layers. Particularly, on top of the second layer, fluence and absorption are in phase until the fluence build up makes it change into counter-phase with the absorption and subsequently overcome it. Fig. 5d shows the corrected estimation and Fig. 5e are the histograms for the orientation of each tendon within the dashed box.

**Fig. 6:**
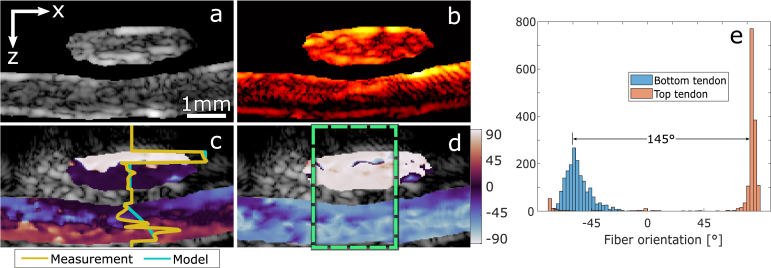
Analysis of tendons with a relative orientation of 30° between them. (a) is the ultrasound image, and (b) is the mean PA image. (c) is the phase detection for the two layers. The profile plot of the phase shows a progressive variation caused by the phase difference between the built-up fluence from the first layer and the absorption being much smaller than 90°. (d) is the corrected detection of fibers’ orientation. (e) shows the histograms for the two tendons within the region indicated by the dashed square.

The second measurement is performed on the two tendons positioned at an angle of 30° between them. In Fig. 6c, as we have observed in the previous case, the phase of the DS-PA signal shows a step transition within the top tendon. However, in the bottom tendon, the phase profile presents a gradual transition that has not been observed in previous measurements. This is because the absolute difference between the fluence phase builds up in the first layer and the dichroism of the second layer is much smaller than 180°; therefore, as illustrated in Fig. 1, the phase of the DS-PA signal will slowly approach the fluence phase as α increases. Fig. 6d shows the corrected estimation of the fiber orientation. Compared to the perpendicular sample, more variations can be observed and an accurate fitting of the data will be more challenging. Fig. 6e shows the histograms for both tendons. We can observe how the bottom layer shows a larger dispersion in the estimated angle. This is due to the lower SNR and increased effect of the scattering.

## 4 Discussion

In this study, we have presented a model and the respective analyzing method for the in-depth estimation of the fiber orientation in fibrous tissue using DS-PAI. The model and the algorithm were first validated on a single-layer porcine tendon placed at different orientations to assess the capability of DS-PAI to determine the fiber orientation in a homogeneous sample and to explore how fluence can affect the detected orientation in depth. We have observed that in a homogeneous fibrous tissue, the DS-PAI signal phase always presents a 90° jump at the depth where the fluence modulation overcomes the dichroism-induced absorption modulation, which corresponds approximately to the inverse of the tissue attenuation. All our DS-PAI measurement results agree well with the actual orientation of the fibers, with an SME of 3.97°.

We also evaluated the proposed methods on stacked tendons at different angles to track variation in fiber orientation in depth, the behavior of the fluence, and its compensation. In the first case, when the fibers were perpendicularly oriented, only step variations of the phase were observed since the amplitude of fluence and absorption were always in phase or counter-phase. In the second scenario, fibers have an orientation of 30° between them. Given that the phase difference between fluence and absorption is less than 90°, the measured phase corresponds to neither absorption nor fluence but progressively switches towards the phase of the fluence as its modulation increases. In both experiments, we observed that signals from deeper areas exhibited higher standard deviations in their histograms. It is due to our simplification of focusing only on ballistic photons, ignoring scattering effects. In more complex samples, other methods like Monte Carlo simulations will be needed to estimate energy variation and light depolarization due to scattering. One drawback of the gaps observed between the tendons is the considerable energy loss caused by reflection and refraction effects at the tissue-water interfaces, which hinders the evaluation of more complex tissue arrangements at greater depths. However, future studies on biological tissue (even with complex structures) are unlikely to encounter this issue, as tissue’s refractive index is generally more homogeneous. This suggests that greater penetration depths than those demonstrated in this study may be achievable.

We have proposed an optimization approach to estimate the measurement parameters in terms of ϕ, ψ, and α. Since we have an underdetermined system, we used physical constraints on a minimization approach to determine the optics axis ϕ. In particular, we assumed the dichroism ratio parameter β as constant for the measured samples. Furthermore, given the in-determination of the phase under certain conditions like α=β, we used pre-defined constraints for these scenarios. To further improve the estimation of the model, we can consider to use the information of the second harmonic corresponding to:

(12) $$\|ℱ\left\{p_{\mathrm{H2}}\right\}\|=\bar{p}\pi \left(\frac{\alpha \beta }{2}\right),$$

(13) $$∠ℱ\left\{p_{\mathrm{H2}}\right\}=\tan^{-1}\left(\frac{\sin\left(2\left(\phi +\psi \right)\right)}{\cos\left(2\left(\phi +\psi \right)\right)}\right)=2\left(\phi +\psi \right),$$

where the phase of the second harmonic corresponds to the sum of the fluence and absorption phases, and the amplitude is proportional to the product of α and β, respectively. However, from our observations and other previous studies, it is clear that the second harmonic has a low SNR and is difficult to detect in the whole sample (refer to Appendix B). In our experiment, we had a relatively low fluence due to beam divergence after exiting the fiber bundle, and consequently, the amplitude of the second harmonic was inadequate for further analysis. Even so, we could effectively estimate fiber orientation in tissue with homogeneous dichroism until 3.2 mm depth. In future works, we will improve our DS-PA setup to achieve a higher SNR and employ the second harmonic information to estimate the optic axis orientation along with the diattenuation magnitude.

To further develop the method into an in-vivo application, it is necessary to consider some additional challenges. Wavelength optimization is required to optimize the trade-off between dichroism amplitude and penetration depth. Furthermore, the proposed approach only detects angular variations in the plane perpendicular to the beam. We employed a transmission-mode imaging setup to ensure the incidence of the beam is orthogonal to the tendon fibers; however, this approach is impractical for many biomedical applications. A potential direction for future research could involve generalizing the definition of absorption modes ($\mu_{a,o}$, $\mu_{a,e}$) for arbitrary light propagation directions, incorporating a more rigorous analysis based on Fresnel equations. Such generalization would enable three-dimensional fiber orientation estimation in reflection-mode imaging by leveraging multiple light sources to reconstruct the volumetric orientation through compounding. Regarding signal processing, employing physics-constrained deep learning techniques can improve the assessment of the DS-PA signal, either by analyzing pixel-wise modulation or image modulation to account for spatial variation of the fluence.

## 5 Conclusion

To conclude, DS-PAI can provide in-depth information on the optics axis of biological tissue. The proposed model describes the behavior of the DS-PA signals and illustrates the effect of fluence and its relevance for a meaningful volumetric assessment of PA signals. In the stacked tendon experiments, we reached an imaging depth of 3.2 mm with a sufficient SNR for processing. Future studies are needed to determine the limits of the techniques and further develop the models to cope with the increased effects of scattering and depolarization with depth.

## CRediT authorship contribution statement

**Camilo Cano:** Writing – original draft, Validation, Methodology, Investigation, Formal analysis, Conceptualization. **Amir Gholampour:** Writing – review & editing, Methodology, Conceptualization. **Marc van Sambeek:** Writing – review & editing, Supervision, Funding acquisition, Conceptualization. **Richard Lopata:** Writing – review & editing, Supervision, Methodology, Funding acquisition, Conceptualization. **Min Wu:** Writing – review & editing, Validation, Supervision, Methodology, Funding acquisition, Formal analysis, Conceptualization.

## Declaration of competing interest

The authors declare that they have no known competing financial interests or personal relationships that could have appeared to influence the work reported in this paper.

## Figures and Tables

**Fig. A.7:**
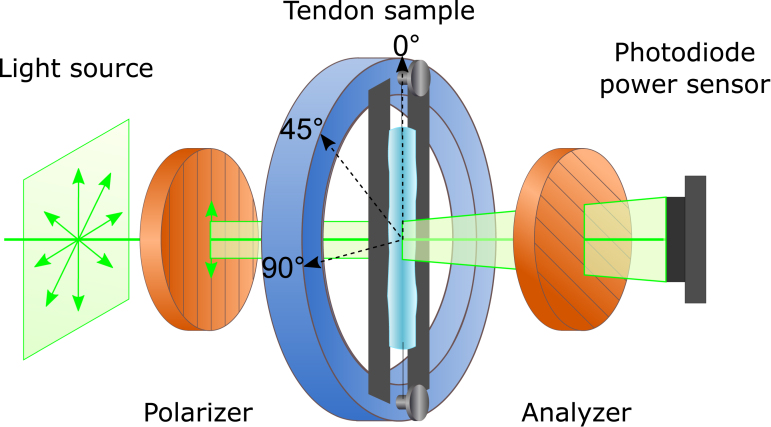
Linear polarimeter for the assessment of light polarization after interacting with a tendon sample.

**Table A.1:** Power measurements for characterization of light polarization.

		Sample orientation		
		0°	45°	90°
Analyzer orientation	0°	4.9 μW	4.3 μW	3.9 μW
	45°	3.7 μW	3.4 μW	3.2 μW
	90°	2.4 μW	2.4 μW	2.4 μW
	135°	4.7 μW	3.4 μW	3.1 μW

**Table A.2:** Polarization parameters.

	Tendon orientation		
	0°	45°	90°
S0 $=I_{H}+I_{V}$	7.3	6.7	6.3
S1 $=I_{H}-I_{V}$	2.5	1.9	1.5
S2 $=I_{P}-I_{M}$	−0.1	0	−0.1
DLP	0.34	0.28	0.24
γ	−1.15°	0°	−1.91°

**Fig. B.9:**
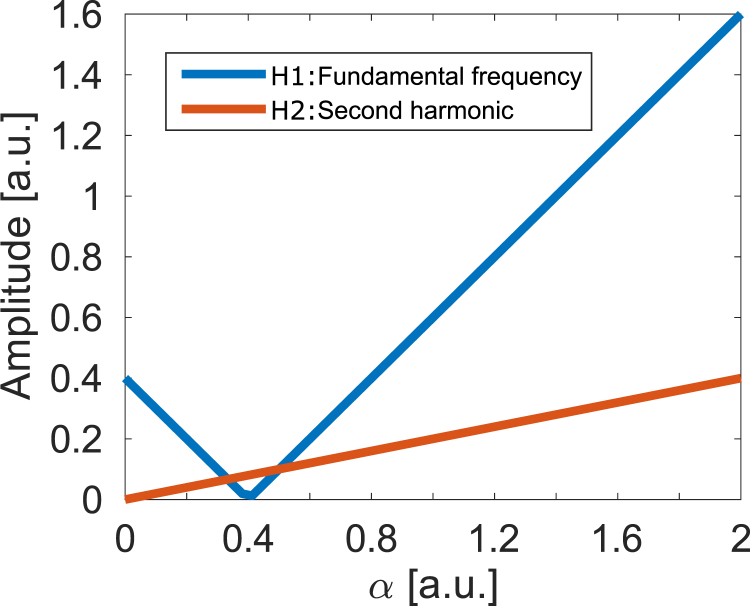
Amplitude of $\|\mathrm{H1}\|/\bar{p}\pi$ and $\|\mathrm{H2}\|/\bar{p}\pi$ as a function of the increase in fluence modulation (α).

## Data Availability

Data will be made available on request.

## Appendix A Assessment of tendon dichroism

To assess the effect of the tendon samples measured in this study on the light polarization, we implemented a linear polarimeter. The sample, a tendon with a thickness of 1.5 mm, is illuminated with vertically polarized light as shown in Fig. A.7, and the transmitted light is analyzed for $I_{H}=0{}^{\circ}$, $I_{P}=45{}^{\circ}$, $I_{V}=90{}^{\circ}$, and $I_{M}=135{}^{\circ}$ to determine the Stokes parameters. Furthermore, we repeated the measurements while positioning the sample (fibers) at an orientation of 0°, 45°, and 90° respect to the vertical axis.

The measurements are found in Table A.1.

In Table A.2, we show the calculated Stokes parameters necessary to assess linear polarization. The parameters are not normalized to observe the transmitted intensity magnitude. DLP $=\sqrt{\left(S_{1}^{2}+S_{2}^{2}\right)}/S_{0}$ is the degree of linear polarization defined between 0 and 1 [25], [27]. $\gamma =tan^{-1}\left(S_{2}/S_{1}\right)/2$ is the angle between the plane of light polarization and the vertical axis [25], [27].

The plane of polarization (γ) barely changes regardless of the orientation of the fibers in the sample, which means that the effects of dichroism and birefringence in the measured samples do not significantly affect the light polarization. However, the intensity ($S_{0}$) decreases depending on the orientation of the fibers, indicating a larger absorption when the fibers are perpendicular to the plane of light polarization. From the difference in intensity between $I_{H}$ and $I_{V}$, we can determine that the normalized dichroism of the tendon is $\Delta \mu_{a}/{\bar{\mu }}_{a}\approx 0.12$. The DLP suggests that around 30% of the light still preserves its polarization after propagating through 1.5 mm of tendon tissue.

## Appendix B Dichroism-sensitive photoacoustic modulation

Fig. B.8a illustrates the normalized DS-PA amplitude as a function of the polarization angle for a representative point at a depth of 1 mm from the sample presented in Fig. 6. Fig. B.8b is the normalized amplitude of the laser output at different polarization angles, which has a mean energy of 5 mJ/pulse. As can be observed, the modulation of the DS-PA signal is at least one order of magnitude larger than the variation in source power. Fig. B.8c 8c is the Fourier spectrum of the signals, where the fundamental frequency *H1* can be clearly detected in the DS-PA signal, while the laser power variation does not reveal any significant modulation at this frequency.

The second harmonic *H2* is less detectable than the fundamental frequency, as can be seen in Fig. B.8c. Additionally, higher frequency components contain noise contributions from other sources, further complicating detection. To understand the difference in amplitude between *H1* and *H2*, let us consider a homogeneous media where the absorption and fluence are in counter phase (cos(2(ϕ−ψ))=−1). Eq. (9) can be rewritten as:

(B.1) $$\|ℱ\left\{p_{\mathrm{H1}}\right\}\|=\bar{p}\pi \beta \left(\frac{\alpha }{\beta }-1\right).$$

Since β<1, we can establish an inequality between the modulation amplitude of the first and second harmonics defined by Eq. (B.1) and Eq. (12), respectively. Eq. (B.2) indicates that, in general, *H1* has a significantly larger magnitude than *H2*.

(B.2) $$\bar{p}\pi \beta \left(\frac{\alpha }{\beta }-1\right)>\bar{p}\pi \left(\frac{\alpha \beta }{2}\right),\;\;\text{for}\;\frac{2\beta }{2+\beta }<\alpha >\frac{2\beta }{2-\beta }.$$

Fig. B.9, illustrates the difference in magnitude between $\|\mathrm{H1}\|/\bar{p}\pi$ and $\|\mathrm{H2}\|/\bar{p}\pi$ as a function of the fluence modulation α, that increases with depth, for a β=0.4. This case exemplifies without loss of generality that *H1* has a higher SNR in most cases than *H2*, making it easier to detect.
